# GPs’ reflections on prescribing addictive hypnotics to older people: a qualitative study

**DOI:** 10.3399/BJGPO.2024.0157

**Published:** 2024-10-30

**Authors:** Holgeir Skjeie, Mette Brekke, Trygve Skonnord

**Affiliations:** 1 General Practice Research Unit, Institute of Health and Society, University of Oslo, Oslo, Norway; 2 Department of General Practice, Institute of Health and Society, University of Oslo, Oslo, Norway

**Keywords:** aged, qualitative research, addictions, general practice, primary healthcare

## Abstract

**Background:**

The European guideline for the diagnosis and treatment of insomnia recommends, for all age groups, only restrictive, short-term, and periodic use of potentially addictive hypnotics. As in other European countries, in Norway, actual practice involving older patients differs substantially from this recommendation, as shown by the persistent high frequency of regular prescriptions of addictive hypnotics.

**Aim:**

To explore experienced Norwegian GPs’ views of the regular prescription of addictive hypnotics to patients aged >70 years living at home.

**Design & setting:**

In-depth individual interviews of a purposive sample of experienced specialists in family medicine at GP offices in Southern Norway.

**Method:**

The interviews used a semi-structured interview guide and were performed between June 2022 and January 2023. Reflexive thematic cross-case analysis was used to analyse the data.

**Results:**

Most of the 11 GPs interviewed had more than 10 older patients who were prescribed hypnotics for daily use and the same number for intermittent prescription. Almost all prescriptions were of z-hypnotics. The GPs knew this was contrary to the guideline. Many were at ease with this fact. They emphasised the need to avoid creating new dependencies. The GPs considered these patients a selected minority within this age group with serious sleep problems, for whom few realistic alternatives were available and whose tolerance over time was better than expected. This logic of pragmatic practice reflected a patient-centred approach and respect for the patient’s view in a shared decision-making process, combined with challenges of limited alternatives and resources.

**Conclusion:**

A 'zero vision' on the prescription of addictive hypnotics to older people may neither be prudent nor realistic in the context of general practice.

## How this fits in

There is a lack of research on the dilemmas of prescribing hypnotics to older people in general practice. In addition, contrary to national guidelines for treating people with insomnia, Norwegian GPs regularly prescribe addictive hypnotics for daily use by older patients living at home. The updated European Insomnia Guideline of 2023 opens the way for off-label use in some cases on an individual basis. This apparent contradiction between national guideline versus practice arises from the GP’s patient-centred approach and shared decision making, and their perception of a lack of realistic alternatives. Recommendations should acknowledge these premises and create better suited methods for the prudent prescription of these drugs in general practice.

## Introduction

A good night’s sleep is essential for all people.^1,2^ However, epidemiological studies have reported that 50% of people aged >60 years report significant sleep problems.^3^ The European guideline for the diagnosis and treatment of insomnia recommends for all age groups restrictive, short-term and/or periodic use of potentially addictive hypnotics and emphasises alternative non-pharmacological interventions and the use of cognitive behavioural therapies.^4^ Long-term use of these drugs is associated with the risk of drug dependency, falls, traffic accidents, and possible cognitive changes.^5–8^ Restrictive use of these drugs, especially for the older population, is recommended because of increased prevalence of multimorbidity and increased risk of frailty.^5,7,9^

General practice in Norway,^10^ as in other European countries,^11–13^ differs substantially from these guideline ideals in that there is a persistent high frequency of regular prescriptions for daily use of addictive hypnotics to the older patient population.^14^ European studies have confirmed and explored how and why reality differs from the guideline recommendations. Regular prescription is acknowledged as professionally problematic by GPs, but this is considered to be difficult to change. Qualitative data suggest various possible explanations, including time pressures, lack of realistic non-pharmacological alternatives, discomfort involved in confrontation with patients, doubt about the validity of the guidelines for individual situations, and sympathy for the patient’s life situation.^12,14–20^

The general practice context ideally implies a long-lasting relationship between the patient and physician. The patient-centred method, introduced in 1986 by Levenstein *et al,* ^21^ and the past decades of increasing emphasis on shared decision making,^22,23^ have become integrated into sound general practice. The French sociologist Pierre Bourdieu (1930–2002) described practice in the social context with its organisational framework and structures of power as a game on a field with a set of written and unwritten rules. This is the *doxa* that creates the premises for the choices of action and, as a consequence of these rules, the *habitus* (the way we act).^24^ Patient-centred practice and shared decision making are part of the doxa of general practice. Their application can help to explain why the actions, habitus, and the guidelines seem so far apart.

There is a lack of qualitative studies on the dilemmas of prescribing addictive hypnotics to older people in general practice. With this background, we explored the views of experienced Norwegian GPs on the perceived dilemmas and choices of action regarding the regular prescription of addictive hypnotics to home-dwelling patients aged >70 years.

## Method

This study used in-depth individual interviews, which is an appropriate research method for exploring sensitive questions of identity, doubt, and conflicting values.

### Data collection

We used a purposive sample of experienced specialists in full-time clinical practice in family medicine who had a high standing among colleagues, were not connected to our university, and were willing to participate in a dialogue on this sensitive topic. Two of the authors (HS and TS), and an academic colleague recruited the 11 participants purposively from four different geographical areas in Southern Norway. No one declined to participate. The interviews used a semi-structured interview guide (Supplementary Information S1) and were performed from June 2022–January 2023. All participants gave informed written consent. They received a gift card of 1500 NOK (110 GBP) for their participation.

### Analysis

We used reflexive thematic analysis, as outlined by Braun and Clarke.^25,26^ We used Bourdieu’s theory of practice as a guiding theoretical lens.^24,27^ All authors (HS, MB, TS) are specialists in family medicine with academic university positions and previous experience in qualitative research. Our epistemological position is that of critical realism.^28^ By this we mean that reality exists as true observable entities, but we acknowledge and interpret the complex and often hidden structures influencing what we observe. HS and MB developed the idea; HS and TS developed the protocol; HS carried out the interviews. The interviews were taped on a portable recorder, and transcribed in verbatim, then erased. Transcripts were secured on computer with only author access. NVivo (version 12) software was used in the analysis. All authors read all interviews and participated in the stages of analysis. Analysis was on semantic content. HS conducted all the interviews. After three interviews, we had a joint meeting on data content, direction, function of interview guide, and eventual redirection and revision. Two of us (TS and HS) then met to discuss and develop initial codes from one chosen interview, as a reflexive guide in the further coding process and development performed by HS. We had continuing correspondence and several joint meetings in the stages of analysis. We searched for themes, and reviewed themes, before finally creating the code and theme tree, naming the themes, and deciding on the outline of the report. The report was written by HS and reviewed by MB and TS. HS carried out a participant validation with three participants, who independently, after reading the Results section, verified the validity of the main themes in a conversation with HS. In the quotes, to preserve anonymity, all participants are identified only by age, gender, and fictitious names. This report follows Standards for Reporting Qualitative Research: a synthesis of recommendations.^29^

## Results

Eleven GPs, all specialists in family medicine, participated in the individual interviews, which lasted 53–70 minutes. All interviews took place in the participant’s office. There were six women, five men, aged 36–65 years, with 8–35 (median 20) years of experience as a GP. All worked in a group practice of 2–7 GPs. Their patient lists comprised 1000–1500 (median 1250) patients, which was higher than the Norwegian mean of 1033 at the time of the interviews. Four GPs worked in rural areas, five in medium-sized towns, and two in a large city. In the reflective thematic cross-case analysis, we developed the following four main themes to shed light on why these experienced clinicians chose to prescribe contrary to the guidelines: 'The basis of confidence'; 'The logic of the prescriptions'; 'The logic of a patient-centred approach'*;* and 'The logic of the rules of practice'. We elaborate on each of these below.

### The basis of confidence

The participants were mostly satisfied with their working life. Many spoke of a meaningful job in a privileged position of patient trust. They described a wide range of daily clinical challenges that they mastered, but they also told about an increasing workload over the years and increasing demands from the patients. However, most participants felt this was within the limits of tolerance. They emphasised that being part of a well-functioning practice was crucial:

*'Yes* [laughter] *... well I am quite okay, and I am part of a very nice medical centre. And I feel I have in a way a good rhythm where the workload is not too heavy and where I am able to have at least that much freedom and self-determination regarding my own everyday working day so that I do not get worn out. I feel it is a lot of work, but I also feel, for my part, that I do not really recognise myself in this* [GP] *crisis*.' (Benjamin, M, 36)

*'It is demanding, and I get tired from work every day. This is how it has been for years, so that is normal. But I feel that I have a very good job*.' (Kristian, M, 65)

A striking feature in the interviews was the participants’ trust in and their reliance on their practice colleagues. Many described a common policy for handling prescriptions of potentially addictive medications, not only for older patients, but also for all patients. They described learning from each other, practice alignment, and support in the handling of issues that were sometimes contrary to guidelines:

*'Because I eat lunch every day with two colleagues and there are no questions too big or too small. You can come with a heart sigh, or you can come with a “darn, today I was dissatisfied with how I handled the situation or what would you have done?”. Our patients see others in the practice when we are away, and we see regularly each other’s patients. So, over the years we develop insight into each other’s prescription practices, and we see that we are by and large in line with each other. And I think that, for me, this is very good colleague support, not sitting alone in a closed room. So that means a lot*.' (Katarina, F, 55)

Most of the participants knew that they were out of line with the current guidelines concerning these prescriptions, but they did not comment on losing their professional autonomy or their self-respect. They seemed confident in the fact that these prescriptions concerned a minority of this patient population and that they were the exceptions:

*'It is a small problem for me, and a small problem for the patients, and I manage to live with that. There are bigger problems than this*.' (Lisa, F, 44)

*'There are probably other things where I feel more inadequate than this thing with zopiclone*.' (Elisabeth, F, 42)

### The logic of the prescriptions

All of these experienced GPs had some, and most of them had more than 10, patients aged >70 years, living at home, who were prescribed potentially addictive hypnotics on a daily basis. They said that the vast majority of prescriptions was for zopiclone, then zolpidem, the z-hypnotics. Most of the participants described the same numbers of patients being prescribed z-hypnotics as needed or as one or two tablets a week:

'*Almost only zopiclone … and a little zolpidem*.' (Katarina, F, 55)

*'I have quite a few who take one every evening, sort of … surely more than 10 people. And then I have many that take as when needed. They have a packet of 30 that lasts three to four months.'* (Astrid, F, 48)

The participants described variations on how the prescription had started, such as taking over previous prescribing from their predecessor, its introduction after a long period of sleeplessness and reduced quality of life, starting during a crisis, or continuing a prescription after a hospital stay. They all emphasised that they focused more on not adding to the numbers of regular prescriptions than prioritising the tapering off of current prescriptions:

*'We have discussed it, and here we are in rare agreement that we will have a little more liberal practice for elderly patients who have an established dependency, but that we try to avoid establishing new ones*.' (Kristian, M, 65)

One issue emphasised by many of the participants was the lack of reliable and realistic alternatives, both non-pharmacological and pharmacological, and no referral possibilities. Elements of cognitive behavioural therapy were sometimes tried, but half-heartedly and with little enthusiasm from either the patients or the doctors, and with little success. Pharmacological alternatives, such as sedating antihistamines, antidepressants, and melatonin, were sometimes prescribed as alternatives, although either the low tolerance and adverse effects or the lack of effect of these medications were noted as a recurring obstacle:

*'Cognitive therapies ... hmm yes. Maybe we should be better at that. It feels like a kind of walk in the desert … and I do not really think I am able to turn them around. You experience that you put in this effort on something where you think that here I have a resourceful woman, good insight, good possibilities: let us do melatonin, stop coffee, no nicotine, have a regular evening walk … blah blah blah, and you make an arrangement with her and really motivate her, and she is all in, right. Then three weeks have passed, and she is back telling you she cannot take it anymore, and says, “You have to give me back my zopiclone”*.' (Katarina, F, 55)

*'I have nothing else to use, as I know of. As you mentioned, mirtazapine is an alternative, and then they should rather not have doxylamine or alimemazine … Hmm … so there is trimipramine and we used it earlier and some are using it, but we have actually been told that they should rather not have either*.' (Susan, F, 64)

### The logic of a patient-centred approach

The participants knew their patients. They had followed them over the years and described the doctor–patient relationship as one of acceptance and dialogue. The GPs knew their patients’ comorbidities, medication lists, level of function, and social context. Most of the patients took the z-hypnotics as their only addictive medication. The GPs noted that the patients’ statement of medication needs for their sleeping problems was influencing prescription policy. They recognised and validated the patients’ perspectives and provided empathy and care in a sometimes difficult daily life situation:

*'But concerning these … what am I supposed to say to the elderly ladies who take half a tablet, or a tablet every evening? Sleep well and have a good life. And they say that “if I do not take it, I become so tired, and I do not manage to function and all that”. Should I not believe them? Should I say, “No you shall not be allowed to sleep because the guidelines say so and so?” I do not have good definite answers on that. Ehm … because the thing is that the patients’ experience is something other than what is written in the guidelines*.' (Lisa, F, 44)

*'So, if the guidelines are right … then we do wrong. But if you work according to patient-centred practice, you may do things that are right but that are not according to guidelines*.' (Thomas, M, 54)

Several participants said that they were surprised by their patients’ stability over the years in the prescription regularity for the addictive hypnotics. For patients given a regular nightly prescription, contrary to expectations of tolerance and loss of effect, they saw little demand for increasing the dosage:

*'Surprisingly many do not* [increase dosage] *… and that I think is … yes, it is ... the guidelines say that there is a need to increase the dosage. But I feel there are many that … Most of them don’t do that*.' (Astrid, F, 48)

The participants also had few examples of observed serious adverse effects. They could not point to cases of falls and fractures, or significant cognitive changes attributed to the regular prescription of hypnotics to the home-dwelling older patients in their practice:

*'But I feel that it goes really well then … they have not suffered a fall and they have not fractured anything, and they have … ehm … not hallucinated or had confusion or … it seems like they have tolerated it better than the guidelines would state … if I can put it this way*.' (Katarina, F, 55)

The arguments about age and respect of the older patients’ autonomous decisions about the risks and benefits were considered valid by many of the participants, and contrasted with the recommendations of a restrictive prescription policy:

*'But then you have these who say, “It is not that dangerous with me because I am so old”, and I have a problem responding because … maybe there is some truth in that …* [laughter].' (Susan, F, 64)

### The logic of the rules of practice

The participants reflected on the operational demands of running their practice: their patients’ needs and demands; the principle of patient-centredness; and the dilemmas of expectation. They noted they had developed their rules of conduct, rules for running a practice, staying in the 'game' (in Bourdieu's analogy), and getting the work done, of which these prescriptions were part:

*'Well, I think you will not last long as a GP by being a square peg in a round hole. That is not how life is in general practice. When you are handling a lot of guidelines at the same time, you make a best common denominator of what we and the patient agree on … right? And that is how it must be. You just do not start that fight because there are often other things in that life that are more important, and larger, and mean more*.' (Thomas, M, 54)

The participants knew the basic outline of the guideline regarding prescription of potential addictive hypnotics for the older population, including the strict rule of limited, short-time use. Still, at times, with some patients, it was prudent to deviate and not try to change or taper off the prescription. Their descriptions of adaptation of the guideline’s ideals to the individual situation was a common feature. The general consideration of the guideline was that it provided sound knowledge to steer by and an anchor for not drifting too far away from good practice, but guidelines were never laws written in stone:

*'But the fact that it is strict makes it easier for me, in a way, not just prescribing over the top and I think it in a way keeps me in line … but I may still break the guideline and prescribe differently, but then at least I should think twice and take an extra round of reasoning*.' (Lisa, F, 44)

Most of participants acknowledged the limited time available for systematic and repeated non-pharmacological suggestions and efforts about tapering patients off medication as an important limiting factor. They noted that the rules of the practice 'game', as well as the running of the daily schedule, had a ticking clock:

*'Yes, it is probably things you should put an effort into, but then it is the time. Prescription renewal comes in connection with the fact that you have 15–20 minutes per patient and then there is control of diabetes, hypertension, cholesterol, yes, the lot … and then this prescription comes at the end. It always comes at the end, when they are in the doorway going out*.' (Elisabeth, F, 42)

## Discussion

### Summary

The 11 participants were all experienced specialists in family medicine employed in full-time daily practice. They were satisfied with their working life, and the collegial support and fellowship were central to this feeling. All had some and most had more than 10 patients who had a regular prescription for these drugs, and the same numbers of patients on an intermittent prescription. Almost all prescriptions were for z-hypnotics. The GPs all knew the principles of the guideline and acknowledged this part of their prescription policy as contrary to the guideline. They were mostly at ease with this fact. Their emphasis was avoiding the creation of new dependencies. They reflected on these patients as a selected minority in this age group who had serious sleep problems. There were few realistic alternatives available, and these patients’ tolerance over time was better than expected. This logic underlying their pragmatic prescription policy was created by their knowledge of the patient’s history, the long-term doctor–patient relationship, the prevailing patient-centred approach, and acknowledgement of their patient’s view in a shared decision-making process, combined with the limited alternatives and logistical challenges of time and resources in running their practices.

### Strengths and limitations

The participants were purposively sampled as experienced specialists employed in family medicine who were willing to reflect in depth on a sensitive issue. The dialogue was supported by a theoretical perspective and focused on one specific challenge in general practice. The participants talked freely for 1 hour with a colleague with similar experiences (HS). As a GP, HS had subject matter knowledge, or field knowledge, as described by Kvale and Brinkmann.^30^ We performed a participant’s validation.^31^ Despite the cross-case analysis, we argue that the information power of our sample, as outlined by Malterud *et al*,^32^ was sufficient. In qualitative interview research, internal validity is always challenged by both confirmation bias — the possibility of interpreting the data to fit one’s preconceptions — and response bias, meaning that the participants tell the interviewer what they think the interviewer would like to hear. These possibilities cannot be excluded. Also, as an extension of the combined confirmation and response biases, the researcher and participant may construct narratives to fit a common pre-understanding or develop a new understanding of how things should be explained. On the other hand, in a reflexive thematic analysis, results are indeed partially constructed in a dialogue between researcher and participant and then between researcher and written text. By acknowledging this, we hopefully have managed to develop a meaningful interpretation of the data of the matter-of-fact dialogues between two equals of the same profession on this small part of clinical practice.

The themes and point of views of our Norwegian participants may not have a general transferability or external validity. The research question was related to the mechanisms underlying the paradox between the guideline and prescription of z-hypnotics to the older people and the tacit knowledge^33^ embedded in general practice about this. To obtain these reflections, we needed a sample of participants with long experience and trust in themselves and the work they do. As such, these reflections and themes may be more like the reflections of an ideal GP sample and not quite like the current situation on the ground in Norway, and in the UK, which experiences vacancies, higher turnover of GPs, and a feeling of increasing pressure and job dissatisfaction. Further, the specifics of Norwegian general practice, such as the strictly personal contractual system based on listing and capitation, normally small group practices and modest patient lists,^34^ may limit the general transferability to GPs currently working in the UK NHS.

### Comparison with existing literature

There is international acknowledgement that the actual prescriptions of benzodiazepine-like hypnotics to older people far exceeds what is to be expected if guidelines are followed.^11–13^ Coteur *et al* in their excellent interview study with Belgian patients, pharmacists, and GPs on addictive hypnotics for insomnia in all age groups,^16^ highlighted the complexity and the multifaceted societal barriers to discontinuation, and also emphasised GPs’ perceived lack of time and lack of success stories regarding non-pharmacological treatment alternatives. Sirdifield *et al,* in their systematic review and metasynthesis of GPs’ experiences regarding benzodiazepine prescription in general in primary care settings (Belgium, USA, Canada, Norway, Slovenia, UK and Australia),^19^ highlighted GPs’ ambivalent attitudes and the inconsistently applied management strategies. We have found no studies in general practice specifically targeting the prescriptions of addictive hypnotics only to otherwise rather well-functioning older patients living at home. This issue is commonly explored in a broader context, either regarding age groups or groups of benzodiazepines. The conclusion is that prescriptions are inappropriate and that doctors feel bad about this.^15,16,19,35^

Interestingly, there has been a change in the European guideline for the diagnosis and treatment of insomnia between 2017 and 2023.^4,36^ The updated European guideline for the diagnosis and treatment of insomnia of August 2023 introduced the possibility of long-term use by stating: *'Longer-term treatment (off-label use) with BZ* [benzodiazepines] *or BZRA* [benzodiazepine receptor agonists]*, either daily or preferably intermittently, may be initiated in some cases, and the advantages and disadvantages need to be discussed on an individual basis.'* ^4^ It seems that this is what the GP participants have done; they have reflected on advantages and disadvantages on an individual basis, given the prevalence of insomnia in this age group,^3^ and the importance of sleep.^1,2^ They have concluded that, for a select few older patients living at home, it is prudent to prescribe daily use of addictive hypnotics. For other patients in this group, prescription for intermittent use may be prudent. The general practice doxa of the patient-centred method and shared decision making creates a Bourdieusian habitus that is logical and might not be inappropriate. We argue, and there is good evidence^34,37^ in support of this, that continuity of care in a patient-centred approach is beneficial in primary care. Although one consequence is that a zero vision for the prescriptions of addictive hypnotics to older people is neither desirable nor achievable.

### Implications for research and practice

Significant sleep problems in the older population are common and lead to reduced quality of life and level of functioning. The use of addictive hypnotics in the older population internationally remains high. There are well-proven non-pharmacological methods, with emphasis on the cognitive-behavioural models,^2^ which are indicated and suitable for clinical practice in various contexts. There may be benefits in monitoring procedures when adverse effects demand discontinuation, and it may be advisable to focus on predictive factors for problematic medication use. However, according to our findings and the revised European guideline for the diagnosis and treatment of insomnia,^4^ off-label prescription of such drugs for the treatment of insomnia may be sound practice for select patients when alternative treatment methods are not feasible. Further research should acknowledge the importance of acceptability of intervention programmes in general practice.^38^ We should strengthen the development of realistic tapering-off methods suited to primary care.^39^ Further research is needed to understand the prudent prescription of these drugs in the various age groups,^38^ and the time pressures and prioritisation encountered in general practice.^40^

## Data

To ensure confidentiality of the participants in the study, the supporting research data are not publicly available. However, data would be made available upon reasonable request to the first author (HS). The raw data are in Norwegian only.
